# LOCATE: a prospective evaluation of the value of Leveraging Ongoing Citation Acquisition Techniques for living Evidence syntheses

**DOI:** 10.1186/s13643-021-01665-x

**Published:** 2021-04-19

**Authors:** Michelle Gates, Sarah A. Elliott, Allison Gates, Meghan Sebastianski, Jennifer Pillay, Liza Bialy, Lisa Hartling

**Affiliations:** 1 Alberta Research Centre for Health Evidence, Department of Pediatrics, University of Alberta, 4-486C Edmonton Clinic Health Academy, 11405-87 Avenue NW, Edmonton, Alberta T6G 1C9 Canada; 2 Alberta Research Centre for Health Evidence, Department of Pediatrics, University of Alberta, 4-488A Edmonton Clinic Health Academy, 11405-87 Avenue NW, Edmonton, Alberta T6G 1C9 Canada; 3 Alberta Research Centre for Health Evidence, Department of Pediatrics, University of Alberta, 4-482C Edmonton Clinic Health Academy, 11405-87 Avenue NW, Edmonton, Alberta T6G 1C9 Canada; 4 Alberta Strategy for Patient-Oriented Research (SPOR) SUPPORT Unit Knowledge Translation Platform, Department of Pediatrics, University of Alberta, 4-486D Edmonton Clinic Health Academy, 11405-87 Avenue NW, Edmonton, Alberta T6H 1C9 Canada; 5 Alberta Research Centre for Health Evidence, Department of Pediatrics, University of Alberta, 4-488D Edmonton Clinic Health Academy, 11405-87 Avenue NW, Edmonton, Alberta T6G 1C9 Canada; 6 Alberta Research Centre for Health Evidence, Department of Pediatrics, University of Alberta, 4-486A Edmonton Clinic Health Academy, 11405-87 Avenue NW, Edmonton, Alberta T6G 1C9 Canada; 7 Alberta Research Centre for Health Evidence, Department of Pediatrics, University of Alberta, 4-472 Edmonton Clinic Health Academy, 11405-87 Avenue NW, Edmonton, Alberta T6G 1C9 Canada

**Keywords:** Living systematic review, Systematic review, Evidence synthesis, Knowledge synthesis, Evidence-based medicine, Methods, Literature searching, Updating

## Abstract

**Background:**

Living systematic reviews (LSRs) can expedite evidence synthesis by incorporating new evidence in real time. However, the methods needed to identify new studies in a timely manner are not well established.

**Objectives:**

To explore the value of complementary search approaches in terms of search performance, impact on results and conclusions, screening workload, and feasibility compared to the reference standard.

**Methods:**

We developed three complementary search approaches for a systematic review on treatments for bronchiolitis: Automated Full Search, PubMed Similar Articles, and Scopus Citing References. These were automated to retrieve results monthly; pairs of reviewers screened the records and commented on feasibility. After 1 year, we conducted a full update search (reference standard). For each complementary approach, we compared search performance (proportion missed, number needed to read [NNR]) and reviewer workload (number of records screened, time required) to the reference standard. We investigated the impact of the new trials on the effect estimate and certainty of evidence for the primary outcomes. We summarized comments about feasibility.

**Results:**

Via the reference standard, reviewers screened 505 titles/abstracts, 24 full texts, and identified four new trials (NNR 127; 12.4 h). Of the complementary approaches, only the Automated Full Search located all four trials; these were located 6 to 12 months sooner than via the reference standard but did not alter the results nor certainty in the evidence. The Automated Full Search was the most resource-intensive approach (816 records screened; NNR 204; 17.1 h). The PubMed Similar Articles and Scopus Citing References approaches located far fewer records (452 and 244, respectively), thereby requiring less screening time (9.4 and 5.2 h); however, each approach located only one of the four new trials. Reviewers found it feasible and convenient to conduct monthly screening for searches of this yield (median 15–65 records/month).

**Conclusions:**

The Automated Full Search was the most resource-intensive approach, but also the only to locate all of the newly published trials. Although the monthly screening time for the PubMed Similar Articles and Scopus Citing Articles was far less, most relevant records were missed. These approaches were feasible to integrate into reviewer work processes.

**Systematic review registration:**

Open Science Framework. 10.17605/OSF.IO/6M28H.

**Supplementary Information:**

The online version contains supplementary material available at 10.1186/s13643-021-01665-x.

## Background

Systematic reviews (SRs) aim to rigorously and transparently synthesize all of the available evidence from primary studies, identify potential biases, and produce a single unbiased conclusion about a particular topic [1, 2]. As the volume of available primary research has grown, SRs have become increasingly large and complex, requiring substantial inputs of time and resources to produce [1, 3, 4]. High-quality SRs can take more than 2 years to complete [4], and the lag between a primary study being published and its subsequent integration into a SR has been estimated to range from 2.5 to 6.5 years [5]. Almost half of SRs are out-of-date within 2 years of publication and therefore provide an incomplete representation of the available evidence [6]. The integrity of healthcare decision-making may be compromised when it relies on outdated SRs whose conclusions may (depending on which studies are missing) no longer be accurate nor valid [7, 8].

Innovative solutions to expedite traditional SR processes are being proposed and evaluated, with the aim of balancing time savings and the high level of rigor that characterizes traditional SRs [9]. The concept of a “living systematic review” (LSR) was first proposed in 2014, with the aim of bridging the evidence-to-practice gap that exists when SRs become out-of-date [10]. In contrast to the static nature of traditional SRs, the approach to LSRs is dynamic, including continuous surveillance of the literature and timely incorporation of new evidence (e.g., within 6 months) [11]. Whereas traditional SRs are published in scientific journals, LSRs are typically housed online, such that updates to the review become available in real time [10]. Currently, available guidance indicates that LSRs may be most appropriate for high-priority topics for which the current evidence is of low-to-very low certainty, and where new evidence that is likely to change practice is accumulating rapidly [11, 12].

The production of a LSR requires the sustained effort of review teams over an extended period of time [10, 11]. At the foundation of LSRs is the commitment to continuous or frequent surveillance of the literature; for example, Cochrane recommends that searches for new research be run at least monthly [13]. To make the timely incorporation of evidence possible, it has been suggested that the production of LSRs be assisted by emerging technologies such as automated database alerts, machine learning, and crowdsourcing [9, 13, 14]. There is increasing interest in the use of abbreviated approaches to locate evidence [15], but the benefits and drawbacks of various approaches and how these may be incorporated into traditional workflows are not well known. Potential complementary search approaches need to be tested to better understand their performance, feasibility of implementation, and how their use may impact the findings of a SR.

For three ongoing complementary search methods and the “reference standard” update approach (i.e., conducting a full update of the original search strategy in all search sources after 1 full year), we evaluated and compared the following: (1a) search performance (proportion of studies missed, precision, sensitivity, number needed to read [i.e., the number of records that need to be screened to locate one included study, NNR], and number of unique included studies retrieved); (1b) the impact of newly identified evidence on the results and certainty of evidence for the primary outcomes; (2a) the screening experience (e.g., logistical challenges, opportunities, successes, and barriers); and (2b) the reviewer workload (screening time).

## Methods

### Study conduct

Methods for this prospective evaluation are reported in an a priori protocol, posted 25 October 2018 on the Open Science Framework, https://osf.io/wxebg/ (doi: 10.17605/OSF.IO/6M28H), and are outlined more briefly below.

### Test systematic review

We tested the proposed complementary search approaches on a SR initiated at our center in 2016 focused on the effectiveness of pharmacologic treatments for the acute management of bronchiolitis (International prospective register of systematic reviews [PROSPERO] registration #CRD42016048625). The SR was chosen to test our LSR approach because (a) the topic is of high clinical priority, (b) there is uncertainty about the most effective treatment [16–18], and (c) new evidence is rapidly emerging (median 7, range 4 to 13 studies per year were included in the SR between 2014 and 2018) that could alter conclusions and/or clinical practice. The primary outcomes of the SR were outpatient rate of admission and inpatient length of stay. Additional file 1: Appendix 1 shows the selection criteria for the SR.

The literature search was developed by a research librarian and peer-reviewed following PRESS guidelines (Additional file 1: Appendix 2) [19]. The search was initially run in October 2016 and updated in May 2018 in the following electronic databases: Ovid MEDLINE, Ovid Embase; Cochrane Central Register of Controlled Trials (CENTRAL) via Wiley Cochrane Library; and CINAHL Plus with Full Text via EBSCOhost (1937 to present; removed in the 2018 search because it previously retrieved no unique included studies). This was supplemented by searches of selected conference proceedings, clinical trials registers, hand-searching reference lists of relevant SRs, and contact with content experts. As of May 2018, the search identified 6999 unique records and the SR included 146 trials.

### Complementary search approaches

We tested three automated search approaches over a 1-year period (referred to as “complementary” approaches), between October 2018 and September 2019: (1) Automated Full Search, (2) PubMed Similar Articles, and (3) Scopus Citing References. A research librarian set up each search such that updates would be received by a central e-mail account on an approximately monthly basis, depending on the functionality of each database. We compared the performance of these strategies to the results of a full search update completed at the end of the 1-year period. We refer to the full search update as the “reference standard.”

#### Automated Full Search

The Automated Full Search was very similar to the reference standard, but was adapted such that MEDLINE and Embase could be searched simultaneously via Ovid (Additional file 1: Appendix 3). We set alerts for Ovid to be received monthly. The timing of alerts for Wiley Cochrane Library cannot be controlled by the user and were received on database reload. We supplemented these searches with a Google alert for clinicaltrials.gov (received “as it happens”) and a monthly alert of the Cochrane Proceedings Citation Index (CPCI) via Clarivate Analytics for conference proceedings.

#### PubMed Similar Articles

We undertook a Similar Articles search in PubMed via National Center for Biotechnology Information (NCBI) Entrez manually each month, as the process could not be automated. The Similar Articles function in PubMed allows users to search for citations related to key “seed” articles chosen by the reviewer [20]. We chose 48 seed articles: 13 key SRs and trials chosen by the SR authors, as well as the 3 largest and 3 most recent trials for each intervention (Additional file 1: Appendix 4). We limited the searches by date (i.e., previous month).

#### Scopus Citing References

We set automated monthly alerts for Citing References in Scopus, using the same 48 seed articles that were used in the PubMed Similar Articles search. The Citing References function in Scopus allows users to view all articles that have cited a particular “seed” article. The Citing References search cannot be restricted by date but the monthly alerts reflected new citations during the previous month.

### Reference management and screening

Following a pilot phase, we assigned a pair of reviewers to the management and screening of records retrieved from each of the search approaches. Pairs were matched for speed and accuracy, based on data collected during the pilot round. We approximated the approach to reference management and screening that may occur in a LSR. One reviewer in each pair received the automated search alerts via e-mail (or ran the search, for PubMed Similar Articles) and forwarded these to the other reviewer in the pair for screening. Duplicate records were not removed. Reviewers screened records independently in duplicate, in a two-phase process (titles and abstracts followed by full texts), and came to agreement on those included after full-text review. Reviewers screened records directly from the e-mail records of the search alerts.

At the end of the 1-year period, a research librarian uploaded the results of the full search update to an Endnote (v.X7, Clarivate Analytics, Philadelphia, PA) library and removed duplicates. The records were transferred to a Microsoft Office Excel (v.2016, Microsoft Corporation, Redmond, WA) spreadsheet for screening. As with the other search approaches, records were screened independently by two reviewers. The final inclusion of studies in the SR was determined by consensus between the two reviewers. This was supplemented by scanning the reference lists of the included studies and pertinent SRs identified by the search.

### Data collection and analysis

#### Search performance

One reviewer documented the following in an Excel spreadsheet each month: the number of records (a) retrieved by the search, (b) screened by title and abstract, (c) reviewed by full text, and (d) included in the SR. As shown in Table 1, for each search approach, we calculated performance metrics using standard formulae, as defined by Cooper et al. [21], and the proportion of studies missed compared to the reference standard.

**Table 1:** Definitions and formulae for the search performance metrics used to evaluate the complementary approaches

Performance metric^a^	Definition and formula
Proportion of studies missed^b^	Number of records not identified by the search, out of the total identified by the reference standard: # relevant studies complementary search approach/# studies included using the reference standard approach × 100
Precision (specificity)	The number of relevant studies identified by the search, relative to the total number of records identified by the search: # relevant studies identified/# records retrieved by the search × 100
Sensitivity	The number of records correctly identified by the search, relative to the total number of relevant studies that exist (identified by the reference standard): # records retrieved by the search/total number of potentially eligible articles that may exist × 100
Number needed to read (NNR)	The number of records identified by the search that need to be screened to locate one included study: 1/precision

^a^Metrics were calculated for each of the complementary search approaches and compared to the reference standard approach

^b^We planned to also record any additional studies located by a complementary method that were not located via the reference standard approach, but this was not applicable

#### Impact on results and certainty of evidence

At the end of the 1 year, one reviewer extracted the following data from studies located via any of the search approaches using a standardized form in Excel: publication characteristics (author, year, country, design, funding source, language), population (age, sex, setting (inpatient or outpatient)), intervention and comparator (drug, dose, timing, duration, mode of administration), co-interventions, and outcome data for the primary outcomes. A second reviewer verified the extraction.

Two reviewers independently assessed the risk of bias of new included studies using the Cochrane Risk of Bias Tool (version 2011) [22]. We assessed trials to be at overall high risk of bias when any critical domain was judged to be at high risk of bias, unclear risk of bias when any critical domain was judged to be at unclear risk of bias and no domain was at high risk of bias, and low risk of bias when there were no concerns in any critical domain. Reviewers resolved disagreements by discussion.

When new included studies were located by any search approach, we added relevant study data to pre-existing pairwise meta-analyses (any of the individual treatments vs. placebo) in Review Manager (RevMan v.5.3, The Nordic Cochrane Centre [Cochrane Collaboration], Copenhagen, Denmark). We pooled data using the Dersimonian and Laird random effects model [23] and present the findings as mean differences (MD) with 95% confidence intervals (CIs). For each new meta-analysis, two reviewers independently appraised the outcome-level certainty of evidence using the Grading of Recommendations Assessment, Development and Evaluation (GRADE) approach [24]. Discrepancies in ratings between the reviewers were resolved by discussion. For ease of interpretation, we present the results of the appraisals in GRADE summary of findings tables and report decisions to rate down the certainty of evidence explicitly. For each complementary search approach, we recorded the timing (i.e., month) at which any changes to our classification of the results and certainty in the evidence occurred.

#### Feasibility and time requirement

Throughout the year, reviewers kept a log of thoughts and experiences related to logistical challenges, opportunities, successes, and barriers in an Excel file. At the end of the 1 year of testing, the reviewers came to consensus on considerations for research groups undertaking LSRs based on their experiences. We had planned to analyze the qualitative data thematically, but given the small amount of data collected, these were instead summarized narratively.

We had initially planned to use a time log in Google forms to collect monthly data related to the search and screening process for each review team, to the closest 5 min per task. At the end of the project, it became apparent that time estimates tended to be overestimated using this method. Thus, we instead assigned a standard time per record for screening, estimated from the time logs (0.5 min per title/abstract; 5 min per full text). This had the advantage of eliminating confounding by differences in the speed of reviewer pairs from our comparison. For each complementary search approach, we calculated descriptive statistics (i.e., medians, ranges) in Excel for the number of hours spent screening per month and over the 1-year period. We retrospectively removed duplicates from the records retrieved via each complementary approach to estimate the number of duplicates screened using each approach.

## Results

Table 2 shows the records retrieved, screened by full text, and included using each search approach across the 1 year of testing (see Additional file 1: Appendix 5 for data by month). Between October 2018 and September 2019, we located 611 records via the reference standard (full search update; 505 after removal of duplicates), screened 24 by full text, and included four new trials in the SR [25–28]. Characteristics of the trials are shown in Additional file 1: Appendix 6.

**Table 2:** Records retrieved and screened and trials included using each of the search approaches

Search approach	Records retrieved and screened by title and abstract	Records screened by full text	Eligible trials^a^	Included trials^b^
Automated Full Search	816	21	7	4
Similar Articles (PubMed)	452	11	1	0
Citing References (Scopus)	244	7	1	1
**Total from complementary approaches**	**1512**	**39**	**9**	**4**
**Reference standard (full search update at 1 year)**	**611 (505 after duplicates removed)**	**24**	**4**	**4**

^a^Eligible trials were those that met the eligibility criteria for the SR

^b^Included studies were those that met the eligibility criteria and had not been previously located by another (or the same) search approach. One study (Chen et al. [25]) was located in the same month by the Automated Full Search and the Scopus Cited References search

### Search performance

Table 3 shows a summary of the search performance metrics for each complementary approach compared to the reference standard. Of the complementary approaches, only the Automated Full Search located all four of the trials that were found using the reference standard full update search. These were located between 6 and 12 months earlier than via the reference standard, in months 1 [27], 5 [28], and 7 [25, 26]. The Automated Full Search also had the best precision among the complementary approaches (0.49%) and therefore the smallest number needed to read (NNR; 204 records). The Scopus Citing References search located only one of the included trials [25], during the same month that it was retrieved by the Automated Full Search. The precision of the search was slightly lower than the Automated Full Search (0.41%) and the NNR correspondingly higher (244 records). Finally, the PubMed Similar Articles search also located only one of the included trials [25] during month 9, after it had already been retrieved via the Automated Full Search and the Scopus Citing References search. This search approach had the lowest precision (0.22%) and highest NNR (455 records).

**Table 3:** Search performance metrics for each of the complementary approaches compared to the reference standard approach

Search approach	Proportion missed (%)	Precision (%)	Sensitivity (%)	Number needed to read
Automated Full Search	0	0.49	100	204
Similar Articles (PubMed)	75	0.22	25	455
Cited References (Scopus)	75	0.41	25	244
Reference standard (full search update at 1 year)	Not applicable	0.79	Not applicable	127

### Impact on the results and certainty of evidence

Three of the newly included studies provided data for inpatient length of stay; two for the analysis of oxygen therapy vs. control [25, 26] and one for hypertonic saline vs. control [27]. One of the newly included studies [28] did not report on any of the primary outcomes. Table 4 shows the GRADE summary of findings for each outcome comparison at baseline (August 2018) and after incorporation of the newly included studies (see Additional file 1: Appendix 7 for forest plots).

**Table 4:** GRADE summary of findings at baseline and after the addition of newly located trials

Timing	RCTs (participants)	Anticipated absolute effects (95% CI) Length of stay, days		***I***^**2**^	Certainty of evidence	Conclusion
		Control (range)	Intervention			
**Oxygen therapy vs. control**						
August 2018 (baseline)	3 (375)	Mean 2.0 to 6.2 days	0.02 more (0.37 fewer to 0.41 more)	0%	Low^a^ ⊕⊕⊝⊝	May be little to no difference
March 2019 (added Chen et al. [25] and Ergul et al. [26])	5 (467)	Mean 2.0 to 6.2 days	0.28 fewer (0.92 fewer to 0.36 more)	54%	Very low^b^ ⊕⊝⊝⊝	Very uncertain
**Hypertonic saline vs. control**						
August 2018 (baseline)	19 (2377)	Mean 1.8 to 7.4 days	0.46 fewer (0.77 fewer to 0.15 fewer)	78%	Moderate^c^ ⊕⊕⊕⊝	Probably reduces
September 2018 (added Morikawa et al. [27])	20 (2505)	Mean 1.8 to 7.4 days	0.43 fewer (0.73 fewer to 0.13 fewer)	77%	Moderate^d^ ⊕⊕⊕⊝	Probably reduces

*CI*, confidence interval; *I*^*2*^, measure of statistical heterogeneity; *RCT*, randomized controlled trial

^a^*Serious concerns about the risk of bias (−1):* Two of the three included studies are at high risk of bias (selection, performance, detection biases); *serious concerns about imprecision (−1):* small sample size (<400) and the 95% confidence interval is wide, including the potential for important benefit and harm; *no serious concerns about indirectness, inconsistency, or other considerations*

^b^*Serious concerns about the risk of bias (−1):* Four of the five included studies are at high risk of bias (selection, performance, detection biases); *serious concerns about imprecision (−1):* the 95% confidence interval is wide, including the potential for important harm; *serious concerns about inconsistency (−1):* some difference in direction of effects (Ergul et al. [26] showing benefit), minimal overlap in confidence intervals for the two more precise studies; *no serious concerns about indirectness or other considerations*

^c^*Serious concerns about inconsistency (−1):* There is heterogeneity in direction and size of effects that cannot be fully explained by a priori subgroup analyses (by the presence of co-interventions in each group), and several confidence intervals are not overlapping. *No serious concerns about the risk of bias, indirectness, imprecision, or other considerations*

^d^*Serious concerns about inconsistency (−1):* There is heterogeneity in direction and size of effects that cannot be fully explained by a priori subgroup analyses (by the presence of co-interventions in each group), and several confidence intervals are not overlapping. *No serious concerns about the risk of bias, indirectness, imprecision, or other considerations*

In August 2018 (baseline), the analysis of length of stay for oxygen therapy vs. control included 3 studies (375 participants), and the pooled estimate showed that there may be little to no difference between groups (MD 0.02 days, 95% CI −0.37 to 0.41, *I*^2^=0%, low certainty). In March 2019, two new trials were added to the analysis. The addition of these studies did not change the conclusion about the effect of oxygen therapy on length of stay, but the certainty of evidence was reduced due to rating down one level for inconsistency (MD −0.28 days, 95% CI −0.92 to 0.36, *I*^2^=54%, 5 RCTs, 467 participants, very low certainty).

At baseline, the analysis for hypertonic saline vs. control included 19 RCTs (2377 participants), and the pooled estimate showed that hypertonic saline probably reduces the length of stay compared to control (MD −0.46 days, 95% CI −0.77 to −0.15, *I*^2^=78%, moderate certainty). The addition of one new trial in September 2018 did not alter the conclusion nor certainty in the evidence (MD −0.43 days, 95% CI −0.73 to −0.13, *I*^2^=77%, 20 RCTs, 2505 participants, moderate certainty).

### Feasibility and reviewer time requirement

Table 5 provides a summary of reviewer workload using each complementary approach. Using the reference standard approach, the total reviewer workload was 12.4 h or 1.6 days (505 titles and abstracts, 24 full texts). Compared to the reference standard approach, the total screening workload was larger (17.1 h or 2.1 days) for the Automated Full Search, but less for the PubMed Similar Articles (9.4 h or 1.2 days) and the Scopus Citing References searches (5.2 h or 0.7 days).

**Table 5:** Reviewer workload for each of the complementary search approaches

Search approach	Records screened by title and abstract	Duplicates screened	Records screened by full text	Screening time, hours (days)^a^
Automated Full Search	816	187	21	17.1 (2.1)
Similar Articles (PubMed)	452	3	11	9.4 (1.2)
Citing References (Scopus)	244	136	7	5.2 (0.7)
**Total from complementary approaches**	**1512**	**326**	**39**	**31.7 (4.5)**
**Reference standard (full search update at 1 year)**	**505**	**0 (removed)**	**24**	**12.4 (1.6)**

^a^Assumed 0.5 min per title/abstract and 5 min per full text for each reviewer and an 8-h workday. This estimate appeared to align with the average time reported by reviewers over the 1-year of screening

Median (range) monthly workload was 65 (39 to 98) titles and abstracts and 1 to 4 full texts for the Automated Full Search (1.4 h); 39 (39 to 49) titles and abstracts and 0 to 2 full texts for PubMed Similar Articles (0.8 h); and 15 (2 to 44) titles and abstracts and 0 to 2 full texts for Scopus Citing References (0.4 h). For both the Automated Full Search (*n* = 187, 23% of total) and Scopus Citing References (*n* = 136, 56% of total), reviewers screened a large number of duplicates. Few duplicates were screened using the PubMed Similar Articles approach (*n* = 3); these appeared to be related to database indexing errors (record corrected and reappeared in a later month).

Overall, the reviewers found it feasible to conduct a monthly screening of this yield and encountered few major challenges related to the search alerts. This was especially the case for the Similar Articles and Citing References searches, as only one database was used for each which made the process straightforward. The Automated Full Search proved to be a bit more difficult because several databases were used which provided alerts at varying frequencies. In 2 months, there were errors in the Ovid alerts, which meant that they needed to be re-run by a librarian prior to screening. It was sometimes difficult to keep on top of all the e-mail alerts, as some databases did not provide updates when no new records were retrieved (it was unclear whether the alert was still functioning). The format of the Ovid alerts could be difficult to read, and it may have been easier to transfer the records to Excel for screening to avoid missing records or having to read all the text. The records from Scopus were also difficult to manage, as reviewers received multiple emails each month (one for each seed article cited), then needed to click separate links to review each record that cited the seed article. For both the Automated Full Search and the Scopus Citing References searches, reviewers noticed that they screened many duplicates. The Cochrane database also provided many records that could have been recently indexed but were several years old (obvious excludes). Finally, although the monthly time commitment was small, it was still necessary to schedule this into the reviewer workload in order to avoid falling behind on screening. This was sometimes difficult, and reviewers found that it would be best to schedule in dedicated time to retrieve and screen records each month.

## Discussion

In this 1-year prospective study, we evaluated the feasibility and utility of three complementary search approaches compared to the reference standard (full update search) in the context of a hypothetical LSR. Via the reference standard, reviewers screened 505 titles/abstracts and 24 full texts and identified four new trials (NNR 127; 12.4 hours) which contributed to two meta-analyses for length of stay (one of two primary outcomes). Of the complementary approaches, only the Automated Full Search, which was the most resource-intensive approach, located all four of these trials. While these trials were located 6 to 12 months sooner than via the reference standard, their addition to the pooled analyses did not change the SR’s conclusion nor the certainty of the evidence for the outcome of interest. The PubMed Similar Articles and Scopus Citing References approaches located far fewer candidate records, thereby requiring less screening time; however, each approach located only one of the four new trials (75% missed). Reviewers found it feasible to conduct monthly screening for searches of this yield (median 15 to 65 records/month). Though the monthly screening load was small, it was necessary to schedule this in among other competing priorities to be sure that it was completed in a timely manner.

Although we chose a SR on a clinically important topic for which the evidence for many treatments was of low-to-very low certainty at the outset, after 1 year, we located relatively little new evidence to incorporate into the SR. Of the newly located evidence, only two small trials contributed to a meta-analysis where our certainty in the effect of treatment was low, and these trials did not alter the conclusion nor improve the certainty of evidence. Our findings highlight one of the values of the LSR approach. Authors of traditional SRs typically run a search update and add relevant primary studies as close as possible to publication to ensure the timeliness of the review. This can be time consuming and inefficient, especially if the findings of the SR are unchanged. One of our main objectives was to test whether the LSR approach improved up-to-dateness by locating and incorporating new findings sooner. In a true LSR, however, authors may develop a priori decision rules to decide when an update of the meta-analysis is needed, based on whether the results of new trials are likely to change the conclusion or certainty in the evidence [11, 13, 29]. In our case, the newly located studies would not need to be added immediately, as they were unlikely to alter the conclusions. In addition to saving time and effort on the part of the review team, such decision rules should be carefully considered at the outset of the LSR, to avoid the potential for the type I error associated with frequent re-analysis each time a new study is found [29].

Based on standard metrics, the performance of each of the complementary approaches was substandard relative to the reference approach. Each of the approaches was substantially less precise, such that reviewers needed to screen more records in order to locate relevant trials. The Similar Articles and Citing References approaches required far less effort on the part of the reviewer (about half or less records to review), but were imprecise and inadequately sensitive, locating only one of the four new trials that should have been included over the 1-year period. The relevance of this finding is unclear, given that the new trials did not contribute to changing the results of conclusions for the outcome of interest. Drawing from evidence on the use of automation technology in SRs, however, trust is highly important to the acceptance of novel or unconventional approaches to SR methodology [30]. Reviewers may not be accepting of approaches that do not locate all of the studies that would be found using traditional approaches. The findings of this study are not adequate to recommend a particular approach. To develop recommendations, there is a need for abbreviated search approaches in the context of LSRs to be further studied over a longer period of time and for a broad array of relevant topics. For example, we located limited guidance on the selection of seed articles for the Similar Articles and Citing References searches, which could have had important impacts on our findings. In the context of true LSRs, it is important that the search approaches be periodically evaluated to ensure an acceptable balance of rigor and efficiency [13].

Another barrier to the adoption of novel practices is the fear of the unknown and assumed lack of compatibility with traditional work practices [30, 31]. A prerequisite to a successful LSR is the availability of a review team that has the expertise, capacity, and motivation to sustain the review over a long period of time [13, 31]. This was the first attempt at replicating LSR processes by our review team, and reviewers found it feasible to manage automated search alerts and screen records monthly. Our screening load was relatively low (<100 records per month); depending on the topic and approaches used, screening load could be much higher [31]. Our group experienced challenges that are not dissimilar to others who have piloted the LSR process [31]. It can be overwhelming to receive and compile multiple automated e-mail alerts per month (Automated Full Search and Scopus Citing References), and careful documentation was needed to ensure that none were missed. It was important to have an information specialist available in the event of database errors that others in the team did not know how to manage. Although the monthly workload was small, it was not always easy to find time for monthly screening among other competing priorities. As suggested by others [31], it is important to have a research coordinator on the project who can help keep the team on top of monthly deadlines and ensure accurate documentation over time.

### Strengths and limitations

This is one of few studies investigating the feasibility and performance of using complementary search approaches in LSRs; our findings may help to inform the evolving guidance for LSRs and future pilots. We tested the complementary search approaches on one SR, and our findings may not be generalizable to other approaches or LSRs. There is a need to further test these approaches (and others) on a variety of LSRs to fully understand which might be most useful and in what circumstances. Our searches were developed by an experienced research librarian; we acknowledge that the comprehensiveness of the searches, search terms and databases used, and familiarity with the databases could have an impact on the effectiveness of search methods. The results of the Similar Articles and Citing References searches were dependent on the chosen seed articles; had another set of seed articles been chosen, the findings may have been different.

## Conclusion

During a 1-year pilot test of three complementary search approaches, we found the Automated Full Search to be the most resource-intensive but also the only approach to locate all of the newly published relevant trials. This approach allowed the review team to update the SR 6 to 12 months sooner than traditional approaches (i.e., full search update after 1 year), though the results and conclusions for the primary outcome were unchanged. Compared with the reference standard, the screening workload (number of records and time commitment) was larger. The PubMed Similar Articles and Scopus Citing References approaches located far fewer candidate records, thereby requiring less screening time; however, each approach located only one of the four new trials (75% missed). Reviewers found it feasible to conduct monthly screening for searches of this yield (median 15 to 65 records/month), but noted minor challenges in fitting the monthly screening workload in among multiple other competing priorities.

## Supplementary Information

**Additional file 1: Appendix 1.** Selection criteria for the systematic review. Description of data: Inclusion and exclusion criteria for the systematic review. **Appendix 2.** Initial search strategy for the systematic review (October 2016). Description of data: Initial search strategy for the systematic review. **Appendix 3.** Strategy for the Automated Full Search. Description of data: Strategy for the automated full search. **Appendix 4.** Seed articles for the PubMed Similar Articles and Scopus Citing References searches. Description of data: List of seed articles for the complementary search approaches. **Appendix 5.** Screening data by month. Description of data: Flow of records through the screening process each month. **Appendix 6.** Characteristics of the four new included trials. Description of data: Characteristics of the included studies. **Appendix 7.** Forest plots for the analysis of length of stay. Description of data: Forest plots for the length of stay analysis.

## Data Availability

The datasets analyzed during the current study are available from the corresponding author on reasonable request.

## Abbreviations

CENTRAL: Cochrane Central Register of Controlled Trials
CI: Confidence interval
CPCI: Cochrane Proceedings Citation Index
GRADE: Grading of Recommendations, Assessment, Development and Evaluations
LSR: Living systematic review
MD: Mean difference
NCBI: National Center for Biotechnology Information
NNR: Number needed to read
PROSPERO: International prospective register of systematic reviews
RCT: Randomized controlled trial
SR: Systematic review
