# Supplementary material for: LOCATE: a prospective evaluation of the value of Leveraging Ongoing Citation Acquisition Techniques for living Evidence syntheses
Source: Syst Rev. 2021 Apr 19;10:116. doi: 10.1186/s13643-021-01665-x (PMC8056603; doi:10.1186/s13643-021-01665-x)
Supplement: Supplementary file 1 — Additional file 1: Appendix 1. Selection criteria for the systematic review. Description of data: Inclusion and exclusion criteria for the systematic review. Appendix 2. Initial search strategy for the systematic review (October 2016). Description of data: Initial search strategy for the systematic review. Appendix 3. Strategy for the Automated Full Search. Description of data: Strategy for the automated full search. Appendix 4. Seed articles for the PubMed Similar Articles and Scopus Citing References searches. Description of data: List of seed articles for the complementary search approaches. Appendix 5. Screening data by month. Description of data: Flow of records through the screening process each month. Appendix 6. Characteristics of the four new included trials. Description of data: Characteristics of the included studies. Appendix 7. Forest plots for the analysis of length of stay. Description of data: Forest plots for the length of stay analysis. [file 13643_2021_1665_MOESM1_ESM.docx]

**Appendix 1.** Selection criteria for the systematic review

**Population**

□ **Include**

Children <2 years (24 months) old experiencing either their first episode of wheeze, or who has been diagnosed with bronchiolitis or respiratory syncytial virus (RSV). Can include studies with ventilated patients if one of the interventions of interest is high-flow oxygen therapy (non-invasive ventilation can include nCPAP).

Note: may include broader age categories if there are results for the <24 month subgroup

□ **Exclude**

Populations ≥2 years (24 months) old; other causes of wheeze (e.g., asthma, general wheezing, other forms of underlying respiratory distress); recurrent wheeze (unless specified it is due to bronchiolitis or RSV); post-bronchiolitis wheeze; pneumonia. Exclude the study if it included participants under invasive ventilator support (e.g., intubation, CPAP, BiPAP).

**Intervention**

□ **Include**:

Any of the following, alone and in combination (note: examples are to assist with screening, others falling under these categories may exist)

Bronchodilators: albuterol, aminophylline, epinephrine/adrenaline, ipratropium, L-epinephrine, Levo-adrenaline, orciprenaline or fenoterol, racemic adrenaline/epinephrine, salbutamol, terbutaline, theophylline

Corticosteroids: adrenal cortex hormones, beclomethasone, betamethasone, budenoside, corticosteroids, dexamethosone, fluticasone, glucocorticoids, hydrocortisone, methylprednisone, prednisolone, prednisone, triamcinoline, triamcinolone

Antibiotics: Amoxicillin, ampicillin, azithromycin, cefdinir, cefuroxime, clarithromycin, erythromycin

Oxygen Therapy: high flow nasal cannula, low flow nasal cannula, high flow oxygen, heated humidified high flow

Hypertonic saline: any saline solution with a concentration of sodium cholride (NaCl) higher than physiologic (0.9%). Common preparations include 2%, 3%, 5%, 7%, and 23% NaCl

Heliox: mixture of helium and oxygen

*can include studies where both treatment and comparison groups are receiving the same co-intervention

□ **Exclude**

Other pharmacologic or non-pharmacologic interventions

**Comparators**

□ **Include**

Placebo, usual care, no treatment, normal saline;

Another intervention of interest, i.e., bronchodilators, corticosteroids, antibiotics, oxygen therapies, hypertonic saline, or heliox (alone or in combination). May include non-invasive CPAP or BiPAP as a comparator to oxygen therapy.

*can include studies where both treatment and comparison groups are receiving the same co-intervention

□ **Exclude**

Any other than those listed above. CPAP or BPAP if not compared to oxygen therapy.

**Outcomes**

□ **Include**

Primary: Outpatient rate of admission, inpatient length of stay

(note, the systematic review also included secondary outcomes that were not of interest to this project)

□ **Exclude**

Any outcome not listed above

**Study design**

□ **Include**

Randomized controlled trials (may be parallel, cross-over, factorial)

□ **Exclude**

Any other study design, non-research, abstracts. Flag relevant systematic reviews to scan reference lists.

**Setting, language, and date of publication**

□ **Include**

Setting: Inpatients or outpatients

Date: any

Language: any

**Appendix 2.** Initial search strategy for the systematic review (October 2016)

**Database:** Ovid MEDLINE(R) Epub Ahead of Print, In-Process & Other Non-Indexed Citations, Ovid MEDLINE(R) Daily and Ovid MEDLINE(R) 1946 to Present

**Date:** 18 Oct 20106

**Records retrieved:** 4,037

| 1. Bronchiolitis/  2. Bronchiolitis, Viral/  3. Metapneumovirus/  4. Parainfluenza Virus 3, Human/  5. Respiratory Syncytial Viruses/  6. Respiratory Syncytial Virus, Human/  7. Respiratory Syncytial Virus Infections/  8. bronchioliti*.tw,kf.  9. ((first* or recurr*) adj3 wheez*).tw,kf.  10. metapneumovirus*.tw,kf.  11. ((para-influenza or parainfluenza) adj virus adj2 ("3" or three)).tw,kf.  12. respiratory s#nc#t#al vir*.tw,kf.  13. RSV.tw,kf.  14. or/1-13 [Combined MeSH and text words for bronchiolitis]  15. Child, Preschool/  16. exp Infant/  17. exp Pediatrics/  18. (baby* or babies or infan* or neo-nat* or neonat* or newborn* or post-matur* or postmatur* or pre-matur* or prematur* or pre-term* or preterm*).tw,jw,kf.  19. (child* or pre-school* or preschool* or toddler*).tw,jw,kf.  20. p?ediatric*.tw,jw,kf.  21. or/15-20 [Combined MeSH and text words for infants]  22. and/14,21 [Combined concept for bronchiolitis and infants]  23. controlled clinical trial.pt.  24. randomized controlled trial.pt.  25. drug therapy.fs.  26. group?.ab.  27. placebo.ab.  28. random*.ab.  29. trial.ab.  30. or/23-29  31. exp animals/ not humans/  32. (animal or animal-model* or animals or bovine or calves or camel* or canine* or cat or cats or dog or dogs or equine or feline or felines or goat* or hamster or hamsters or llama* or mice or monkey or monkeys or mouse or pig or piglet or piglets or pigs or porcine or primate* or rabbit or rabbits or rat or rats or rodent or rodents or sheep or simian* or swine or swines).ti.  33. 30 not (31 or 32) [Modified Cochrane Highly Sensitive search strategy for identifying randomized trials - http://handbook.cochrane.org/]  34. and/22,33 [RCT filter applied]  35. remove duplicates from 34 |
| --- |

**Database:** Ovid Embase 1974 to 2016 Week 42

**Date:** 19 Oct 2016

**Records retrieved:** 2,623

| 1. bronchiolitis/  2. human metapneumovirus/  3. human parainfluenza virus 3/  4. exp human respiratory syncytial virus/  5. viral bronchiolitis/  6. bronchioliti*.tw,kw.  7. ((first* or recurr*) adj3 wheez*).tw,kw.  8. metapneumovirus*.tw,kw.  9. ((para-influenza or parainfluenza) adj virus adj2 ("3" or three)).tw,kw.  10. respiratory s#nc#t#al vir*.tw,kw.  11. RSV.tw,kw.  12. or/1-11 [Combined Emtree and text words for bronchiolitis]  13. exp infant/  14. exp pediatrics/  15. preschool child/  16. toddler/  17. (baby* or babies or infan* or neo-nat* or neonat* or newborn* or post-matur* or postmatur* or pre-matur* or prematur* or pre-term* or preterm*).tw,jx,kw.  18. (child* or pre-school* or preschool* or toddler*).tw,jx,kw.  19. p?ediatric*.tw,jx,kw.  20. or/13-19 [Combined Emtree and text words for infants]  21. and/12,20 [Combined concept for bronchiolitis and infants]  22. crossover procedure/  23. clinical trial/  24. double blind procedure/  25. placebo/  26. prospective study/  27. randomization/  28. randomized controlled trial/  29. single blind procedure/  30. (allocat* adj2 random*).tw.  31. double blind*.tw.  32. placebo*.tw.  33. randomi?ed controlled trial*.tw.  34. RCT.tw.  35. single blind*.tw.  36. ((treble or triple) adj blind*).tw.  37. or/22-36  38. abstract report/  39. case study/  40. letter/  41. (case adj (report* or stud*)).ti.  42. or/38-41  43. 37 not 42  44. exp animals/ not exp humans/  45. (animal or animal-model* or animals or bovine or calves or camel* or canine* or cat or cats or dog or dogs or equine or feline or felines or goat* or hamster or hamsters or llama* or mice or monkey or monkeys or mouse or pig or piglet or piglets or pigs or porcine or primate* or rabbit or rabbits or rat or rats or rodent or rodents or sheep or simian* or swine or swines).ti.  46. 43 not (44 or 45) [Modified SIGN Embase RCT filter - http://www.sign.ac.uk/methodology/filters.html#random]  47. and/21,46 [RCT filter applied]  48. remove duplicates from 47 |
| --- |

**Database:** Cochrane Central Register of Controlled Trials (CENTRAL) via Wiley Cochrane Library (inception to present)

**Date:** 19 Oct 2016

**Records retrieved:** 970

| 1. [mh ^Bronchiolitis]  2. [mh ^"Bronchiolitis, Viral"]  3. [mh ^Metapneumovirus]  4. [mh ^"Parainfluenza Virus 3, Human"]  5. [mh ^"Respiratory Syncytial Viruses"]  6. [mh ^"Respiratory Syncytial Virus, Human"]  7. [mh ^"Respiratory Syncytial Virus Infections"]  8. bronchioliti*:ti,ab,kw  9. ((first* or recurr*) near/3 wheez*):ti,ab,kw  10. metapneumovirus*:ti,ab,kw  11. ((para-influenza or parainfluenza) near/1 virus near/2 ("3" or three)):ti,ab,kw  12. "respiratory s?nc?t?al vir*":ti,ab,kw  13. RSV:ti,ab,kw  14. ^1-#13^  15. [mh ^"Child, Preschool"]  16. [mh Infant]  17. [mh Pediatrics]  18. (baby* or babies or infan* or neo-nat* or neonat* or newborn* or post-matur* or postmatur* or pre-matur* or prematur* or pre-term* or preterm*):ti,ab,kw,so  19. (child* or pre-school* or preschool* or toddler*):ti,ab,kw,so  20. p*diatric*:ti,ab,kw,so  21. ^18-#20^  22. #14 AND #21  23. #14 AND #21 in Trials |
| --- |

**Database:** CINAHL Plus with Full Text via EBSCOhost (1937 to the present)

**Date:** 19 Oct 2016

**Records retrieved:** 254

| S1. (MH "Bronchiolitis")  S2. (MH "Respiratory Syncytial Virus Infections")  S3. (MH "Respiratory Syncytial Viruses")  S4. bronchioliti*  S5. ((first* or recurr*) N3 wheez*)  S6. metapneumovirus*  S7. ((para-influenza or parainfluenza) N1 virus N2 ("3" or three))  S8. "respiratory s?nc?t?al vir*"  S9. RSV  S10. S1 OR S2 OR S3 OR S4 OR S5 OR S6 OR S7 OR S8 OR S9  S11. (MH "Child, Preschool")  S12. (MH "Infant+")  S13. (MH "Pediatrics+")  S14. (baby* or babies or infan* or neo-nat* or neonat* or newborn* or post-matur* or postmatur* or pre-matur* or prematur* or pre-term* or preterm*)  S15. (child* or pre-school* or preschool* or toddler*)  S16. (p#ediatric or p#ediatrics)  S17. S11 OR S12 OR S13 OR S14 OR S15 OR S16  S18. S10 AND S17  S19. PT clinical trial  S20. (MH "Clinical Trials+")  S21. (MH "Placebos")  S22. (MH "Quantitative Studies")  S23. (MH "Random Assignment")  S24. (allocat* N1 random*)  S25. (blind* N1 (doubl* OR singl* OR trebl* OR tripl*)  S26. (clinic* N1 trial*)  S27. (mask* N1 (doubl* OR singl* OR trebl* OR tripl*)  S28. placebo*  S29. "randomi?ed controlled trial"  S30. S19 OR S20 OR S21 OR S22 OR S23 OR S24 OR S25 OR S26 OR S27 OR S28 OR S29  S31. ((MH "Vertebrates+") NOT MH Human)  S32. TI(animal or animal-model* or animals or bovine or calves or camel* or canine* or cat or cats or dog or dogs or equine or feline or felines or goat* or hamster or hamsters or llama* or mice or monkey or monkeys or mouse or pig or piglet or piglets or pigs or porcine or primate* or rabbit or rabbits or rat or rats or rodent or rodents or sheep or simian* or swine or swines)  S33. S30 NOT (S31 OR S32) [Modified SIGN CINAHL RCT filter - http://www.sign.ac.uk/methodology/filters.html#random  S34. S18 AND S33 [RCT filter applied] |
| --- |

**Trial Registry:** ClinicalTrials.gov (<https://clinicaltrials.gov/>)

**Date:** 20 Oct 2016

**Records retrieved:** 59

| Advanced Search >  Study Type: Interventional Studies  Age: Child  Conditions: "Bronchiolitis" OR "Bronchiolitis, Viral" OR "respiratory syncytial virus" OR "respiratory syncytial viruses" OR RSV  First Received: From 01/01/2014 to 10/20/2016 |
| --- |

**Trial Registry:** World Health Organization International Clinical Trials Registry Platform (WHO ICTRP) (<http://apps.who.int/trialsearch/>)

**Date:** 10 Nov 2016

**Records retrieved:** 103

| Advanced Search >  Search for clinical trials in children  Recruitment status is: ALL  Conditions: bronchiolitis OR respiratory syncytial virus OR respiratory syncytial viruses OR RSV  Date of registration: From 01/01/2014 to 10/11/2016 |
| --- |

**Appendix 3.** Strategy for the Automated Full Search

**Database:** Ovid MEDLINE(R) Epub Ahead of Print, In-Process & Other Non-Indexed Citations, Ovid MEDLINE(R) Daily and Ovid MEDLINE(R) <1946 to Present>, Embase <1980 to 2018 Week 18>

**Alert frequency:** Monthly on the first day of each month

| 1 Bronchiolitis/ (14761)  2 Bronchiolitis, Viral/ (2538)  3 Respiratory Syncytial Virus Infections/ (7008)  4 bronchioliti*.tw,kf. (24388)  5 ((first* or recurr*) adj3 wheez*).tw,kf. (3920)  6 respiratory syncytial virus*.tw,kf. (25932)  7 or/1-6 [Combined MeSH & text words for bronchiolitis] (55085)  8 Child, Preschool/ (1270287)  9 exp Infant/ (2026944)  10 (baby* or babies or infan* or neo-nat* or neonat* or newborn* or post-matur* or postmatur* or pre-matur* or prematur* or pre-term* or preterm*).tw,jw,kf. (1756404)  11 (child* or pre-school* or preschool* or toddler*).tw,jw,kf. (2921238)  12 or/8-11 [Combined MeSH and text words for infants] (5181917)  13 and/7,12 [Population concept of bronchiolitis in infants] (27591)  14 controlled clinical trial.pt. (92372)  15 randomized controlled trial.pt. (459781)  16 drug therapy.fs. (5554221)  17 group?.ab. (7264809)  18 placebo.ab. (446334)  19 random*.ab. (2197074)  20 trial.ab. (1026618)  21 or/14-20 (13220061)  22 exp animals/ not humans/ (15420179)  23 (animal* or bovine* or calves or camel* or canine* or cat or cats or chimp* or dog or dogs or equine* or feline* or goat* or hamster* or llama* or mice* or monkey* or mouse* or pig or piglet* or pigs or porcine* or primate* or rabbit* or rat or rats or rodent* or sheep* or simian* or swine*).ti. (4332481)  24 21 not (22 or 23) [Modified Cochrane Highly Sensitive search strategy for identifying randomized trials - http://handbook.cochrane.org/] (8989521)  25 and/13,24 [RCT filter applied] (8368)  26 25 use ppez (4098)  27 bronchiolitis/ (14761)  28 exp human respiratory syncytial virus/ (4914)  29 viral bronchiolitis/ (2881)  30 bronchioliti*.tw,kw. (25049)  31 ((first* or recurr*) adj3 wheez*).tw,kw. (3931)  32 respiratory syncytial vir*.tw,kw. (26361)  33 or/27-32 [Combined Emtree & text words for bronchiolitis] (55680)  34 exp infant/ (2026944)  35 preschool child/ (1402762)  36 toddler/ (3384)  37 (baby* or babies or infan* or neo-nat* or neonat* or newborn* or post-matur* or postmatur* or pre-matur* or prematur* or pre-term* or preterm*).tw,jx,kw. (1731268)  38 (child* or pre-school* or preschool* or toddler*).tw,jx,kw. (2852032)  39 or/34-38 [Combined Emtree and text words for infants] (5178581)  40 and/33,39 [Population concept of bronchiolitis in infants] (27856)  41 crossover procedure/ (55141)  42 clinical trial/ (1474296)  43 double blind procedure/ (146388)  44 placebo/ (310268)  45 prospective study/ (915258)  46 randomization/ (171881)  47 randomized controlled trial/ (956984)  48 single blind procedure/ (31185)  49 (allocat* adj2 random*).tw. (65735)  50 double blind*.tw. (319367)  51 placebo*.tw. (461641)  52 randomi?ed controlled trial*.tw. (317639)  53 RCT.tw. (44161)  54 single blind*.tw. (36384)  55 ((treble or triple) adj blind*).tw. (1411)  56 or/41-55 (3241436)  57 abstract report/ (66795)  58 case study/ (1930150)  59 letter/ (1953295)  60 (case adj (report* or stud*)).ti. (565548)  61 or/57-60 (4132604)  62 56 not 61 (3165638)  63 exp animals/ not exp humans/ (8820102)  64 (animal* or bovine* or calves or camel* or canine* or cat or cats or chimp* or dog or dogs or equine* or feline* or goat* or hamster* or llama* or mice* or monkey* or mouse* or pig or piglet* or pigs or porcine* or primate* or rabbit* or rat or rats or rodent* or sheep* or simian* or swine*).ti. (4332481)  65 62 not (63 or 64) [Modified SIGN Embase RCT filter - http://www.sign.ac.uk/methodology/filters.html#random] (3011195)  66 and/40,65 [RCT filter applied] (4546)  67 66 use emez (2799)  68 or/26,67 [Combined Medline & Embase searches for auto-alert] (6897)* |
| --- |

### * Note: may not allow automated de-duplication as results >6K

**Database:** Wiley Cochrane Library

**Alert frequency:** On database reload

| #1 [mh ^Bronchiolitis] 202  #2 [mh ^"Bronchiolitis, Viral"] 110  #3 [mh ^"Respiratory Syncytial Virus Infections"] 259  #4 bronchioliti*:ti,ab,kw 994  #5 ((first* or recurr*) near/3 wheez*):ti,ab,kw 254  #6 "respiratory syncytial vir*":ti,ab,kw 620  #7 ^19-#6^ 1602  #8 [mh ^"Child, Preschool"] 114  #9 [mh Infant] 15842  #10 (baby* or babies or infan* or (neo next nat*) or neonat* or newborn* or (post next matur*) or postmatur* or (pre next matur*) or prematur* or (pre next term*) or preterm*):ti,ab,kw,so 66269  #11 (child* or (pre next school*) or preschool* or toddler*):ti,ab,kw,so 112592  #12 ^20-#11^ 148049  #13 #7 and #12 1250  #14 #7 and #12 in Trials 1113 |
| --- |

**Other source:** ClinicalTrials.gov

**Alert interface:** Google Alerts (<https://www.google.ca/alerts>)

**Alert frequency:** As it happens

| (bronchiolitis) (clinicaltrials) |
| --- |

**Other source:** Conference Proceedings Citation Index

**Alert interface:** Clarivate Analytics

**Alert frequency:** Monthly

| # 7 | [**197**](http://apps.webofknowledge.com.login.ezproxy.library.ualberta.ca/summary.do?product=WOS&doc=1&qid=7&SID=6Bh6ggTXawpS7YHqGef&search_mode=AdvancedSearch&update_back2search_link_param=yes) | #5 NOT #6  *Indexes=CPCI-S Timespan=All years* |
| --- | --- | --- |
| # 6 | [**251,153**](http://apps.webofknowledge.com.login.ezproxy.library.ualberta.ca/summary.do?product=WOS&doc=1&qid=6&SID=6Bh6ggTXawpS7YHqGef&search_mode=AdvancedSearch&update_back2search_link_param=yes) | TI=(animal* or bovine* or calves or camel* or canine* or cat or cats or chimp* or dog or dogs or equine* or feline* or goat* or hamster* or llama* or mice* or monkey* or mouse* or pig or piglet* or pigs or porcine* or primate* or rabbit* or rat or rats or rodent* or sheep* or simian* or swine*)  *Indexes=CPCI-S Timespan=All years* |
| # 5 | [**203**](http://apps.webofknowledge.com.login.ezproxy.library.ualberta.ca/summary.do?product=WOS&doc=1&qid=5&SID=6Bh6ggTXawpS7YHqGef&search_mode=AdvancedSearch&update_back2search_link_param=yes) | #3 AND #4  *Indexes=CPCI-S Timespan=All years* |
| # 4 | [**663,991**](http://apps.webofknowledge.com.login.ezproxy.library.ualberta.ca/summary.do?product=WOS&doc=1&qid=4&SID=6Bh6ggTXawpS7YHqGef&search_mode=AdvancedSearch&update_back2search_link_param=yes) | TS=("double blind*" or crossover* or group* or placebo* or random* or RCT or "single blind*" or trial* or "triple blind*")  *Indexes=CPCI-S Timespan=All years* |
| # 3 | [**866**](http://apps.webofknowledge.com.login.ezproxy.library.ualberta.ca/summary.do?product=WOS&doc=1&qid=3&SID=6Bh6ggTXawpS7YHqGef&search_mode=AdvancedSearch&update_back2search_link_param=yes) | #1 AND #2  *Indexes=CPCI-S Timespan=All years* |
| # 2 | [**166,565**](http://apps.webofknowledge.com.login.ezproxy.library.ualberta.ca/summary.do?product=WOS&doc=1&qid=2&SID=6Bh6ggTXawpS7YHqGef&search_mode=AdvancedSearch&update_back2search_link_param=yes) | TS=(baby* or babies or child* or infan* or "neo-nat*" or neonat* or newborn* or "post-matur*" or postmatur* or "pre-matur*" or prematur* or "pre-school*" or preschool* or "pre-term*" or preterm* or toddler*)  *Indexes=CPCI-S Timespan=All years* |
| # 1 | [**2,382**](http://apps.webofknowledge.com.login.ezproxy.library.ualberta.ca/summary.do?product=WOS&doc=1&qid=1&SID=6Bh6ggTXawpS7YHqGef&search_mode=AdvancedSearch&update_back2search_link_param=yes) | TS=(bronchioliti* or ((first* or recurr*) NEAR/3 wheez*) or "respiratory syncytial virus*")  *Indexes=CPCI-S Timespan=All years* |

**Appendix 4.** Seed articles for the PubMed Similar Articles and Scopus Citing References searches

General Papers/Treatment Overviews (n = 4 review articles)

Petrarca L, Jacinto T, Nenna R. The treatment of acute bronchiolitis: past, present and future. Breathe (Sheff). 2017 Mar;13(1):e24-e26. doi: 10.1183/20734735.000717. PubMed PMID: 29158779; PubMed Central PMCID: PMC5685214.

Ricci V, Delgado Nunes V, Murphy MS, Cunningham S; Guideline Development Group and Technical Team. Bronchiolitis in children: summary of NICE guidance. BMJ. 2015 Jun 2;350:h2305. doi: 10.1136/bmj.h2305. PubMed PMID: 26037525.

Wohl ME, Chernick V. Treatment of acute bronchiolitis. N Engl J Med. 2003 Jul 3;349(1):82-3. PubMed PMID: 12840097.

King VJ, Viswanathan M, Bordley WC, Jackman AM, Sutton SF, Lohr KN, Carey TS. Pharmacologic treatment of bronchiolitis in infants and children: a systematic review. Arch Pediatr Adolesc Med. 2004 Feb;158(2):127-37. Review. PubMed PMID: 14757604.

Records by treatment:

Bronchodilators

**Systematic reviews, n = 2**

Hartling L, Fernandes RM, Bialy L, Milne A, Johnson D, Plint A, Klassen TP, Vandermeer B. Steroids and bronchodilators for acute bronchiolitis in the first two years of life: systematic review and meta-analysis. BMJ. 2011 Apr 6;342:d1714. doi: 10.1136/bmj.d1714. Review. PubMed PMID: 21471175; PubMed Central PMCID: PMC3071611.

Gadomski AM, Scribani MB. Bronchodilators for bronchiolitis. Cochrane Database Syst Rev. 2014 Jun 17;(6):CD001266. doi: 10.1002/14651858.CD001266.pub4. Review. PubMed PMID: 24937099.

**RCTs, n = 6**

Flores-González JC, Dominguez-Coronel MT, Matamala Morillo MA, Aragón Ramírez M, García Ortega RM, Dávila Corrales FJ, García Palacios MV, Perez Guerrero JJ, García García L, Lechuga Sancho AM. Does nebulized epinephrine improve the efficacy of hypertonic saline solution in the treatment of hospitalized moderate acute bronchiolitis? A double blind, randomized clinical trial. Minerva Pediatr. 2016 Apr;68(2):81-8. Epub 2014 Sep 29. PubMed PMID: 25263242.

Zamani MA, Movahhedi M, Nourbakhsh SM, Ganji F, Rafieian-Kopaei M, Mobasheri M, Khoshdel A, Etemadifar S, Shirani M, Keivani Hafshejani Z. Therapeutic effects of Ventolin versus hypertonic saline 3% for acute bronchiolitis in children. Med J Islam Repub Iran. 2015 May 6;29:212. eCollection 2015. PubMed PMID: 26478870; PubMed Central PMCID: PMC4606955.

Kose M, Ozturk MA, Poyrazoğlu H, Elmas T, Ekinci D, Tubas F, Kurt T, Goktas MA. The efficacy of nebulized salbutamol, magnesium sulfate, and salbutamol/magnesium sulfate combination in moderate bronchiolitis. Eur J Pediatr. 2014 Sep;173(9):1157-60. doi: 10.1007/s00431-014-2309-3. Epub 2014 Apr 2. PubMed PMID: 24687251.

Plint AC, Johnson DW, Patel H, Wiebe N, Correll R, Brant R, Mitton C, Gouin S, Bhatt M, Joubert G, Black KJ, Turner T, Whitehouse S, Klassen TP; Pediatric Emergency Research Canada (PERC). Epinephrine and dexamethasone in children with bronchiolitis. N Engl J Med. 2009 May 14;360(20):2079-89. doi: 10.1056/NEJMoa0900544. PubMed PMID: 19439742.

Walsh P, Caldwell J, McQuillan KK, Friese S, Robbins D, Rothenberg SJ. Comparison of nebulized epinephrine to albuterol in bronchiolitis. Acad Emerg Med. 2008 Apr;15(4):305-13. doi: 10.1111/j.1553-2712.2008.00064.x. PubMed PMID: 18370982; PubMed Central PMCID: PMC2613253.

Skjerven HO, Hunderi JO, Brügmann-Pieper SK, Brun AC, Engen H, Eskedal L, Haavaldsen M, Kvenshagen B, Lunde J, Rolfsjord LB, Siva C, Vikin T, Mowinckel P, Carlsen KH, Lødrup Carlsen KC. Racemic adrenaline and inhalation strategies in acute bronchiolitis. N Engl J Med. 2013 Jun 13;368(24):2286-93. doi: 10.1056/NEJMoa1301839. PubMed PMID: 23758233.

Corticosteroids

**Systematic reviews, n = 3**

Fernandes RM, Hartling L. Glucocorticoids for acute viral bronchiolitis in infants and young children. JAMA. 2014 Jan 1;311(1):87-8. doi: 10.1001/jama.2013.284921. PubMed PMID: 24381968.

Garrison MM, Christakis DA, Harvey E, Cummings P, Davis RL. Systemic corticosteroids in infant bronchiolitis: A meta-analysis. Pediatrics. 2000 Apr;105(4):E44. PubMed PMID: 10742365.

Fernandes RM, Bialy LM, Vandermeer B, Tjosvold L, Plint AC, Patel H, Johnson DW, Klassen TP, Hartling L. Glucocorticoids for acute viral bronchiolitis in infants and young children. Cochrane Database Syst Rev. 2013 Jun 4;(6):CD004878. doi: 10.1002/14651858.CD004878.pub4. Review. PubMed PMID: 23733383.

**RCTs, n = 6**

Lan WP, Wang J, Dai CL, Pan JH. [Efficacy of fluticasone propionate aerosol versus budesonide suspension in treatment of recurrent wheezing caused by bronchiolitis]. Zhongguo Dang Dai Er Ke Za Zhi. 2016 Apr;18(4):316-9. Chinese. PubMed PMID: 27097575.

Jartti T, Nieminen R, Vuorinen T, Lehtinen P, Vahlberg T, Gern J, Camargo CA Jr, Ruuskanen O. Short- and long-term efficacy of prednisolone for first acute rhinovirus-induced wheezing episode. J Allergy Clin Immunol. 2015 Mar;135(3):691-8.e9. doi: 10.1016/j.jaci.2014.07.001. Epub 2014 Aug 13. PubMed PMID: 25129681.

Mesquita M, Castro-Rodríguez JA, Heinichen L, Fariña E, Iramain R. Single oral dose of dexamethasone in outpatients with bronchiolitis: a placebo controlled trial. Allergol Immunopathol (Madr). 2009 Mar-Apr;37(2):63-7. PubMed PMID: 19445861.

Corneli HM, Zorc JJ, Mahajan P, Shaw KN, Holubkov R, Reeves SD, Ruddy RM, Malik B, Nelson KA, Bregstein JS, Brown KM, Denenberg MN, Lillis KA, Cimpello LB, Tsung JW, Borgialli DA, Baskin MN, Teshome G, Goldstein MA, Monroe D, Dean JM, Kuppermann N. Bronchiolitis Study Group of the Pediatric Emergency Care Applied Research Network (PECARN). A multicenter, randomized, controlled trial of dexamethasone for bronchiolitis. N Engl J Med. 2007 Jul 26;357(4):331-9. Erratum in: N Engl J Med. 2008 Oct 30;359(18):1972.. Majahan, Prashant [corrected to Mahajan, Prashant]. PubMed PMID: 17652648.

Alansari K, Sakran M, Davidson BL, Ibrahim K, Alrefai M, Zakaria I. Oral dexamethasone for bronchiolitis: a randomized trial. Pediatrics. 2013 Oct;132(4):e810-6. doi: 10.1542/peds.2012-3746. Epub 2013 Sep 16. PubMed PMID: 24043283.

Teeratakulpisarn J, Limwattananon C, Tanupattarachai S, Limwattananon S, Teeratakulpisarn S, Kosalaraksa P. Efficacy of dexamethasone injection for acute bronchiolitis in hospitalized children: a randomized, double-blind, placebo-controlled trial. Pediatr Pulmonol. 2007 May;42(5):433-9. PubMed PMID: 17394255.

Antibiotics

**Systematic reviews, n = 1**

Farley R, Spurling GK, Eriksson L, Del Mar CB. Antibiotics for bronchiolitis in children under two years of age. Cochrane Database Syst Rev. 2014 Oct 9;(10):CD005189. doi: 10.1002/14651858.CD005189.pub4. Review. PubMed PMID: 25300167.

**RCTs, n = 6**

Tahan F, Ozcan A, Koc N. Clarithromycin in the treatment of RSV bronchiolitis: a double-blind, randomised, placebo-controlled trial. Eur Respir J. 2007 Jan;29(1):91-7. Epub 2006 Oct 18. PubMed PMID: 17050564.

Pinto LA, Pitrez PM, Luisi F, de Mello PP, Gerhardt M, Ferlini R, Barbosa DC, Daros I, Jones MH, Stein RT, Marostica PJ. Azithromycin therapy in hospitalized infants with acute bronchiolitis is not associated with better clinical outcomes: a randomized, double-blinded, and placebo-controlled clinical trial. J Pediatr. 2012 Dec;161(6):1104-8. doi: 10.1016/j.jpeds.2012.05.053. Epub 2012 Jun 28. PubMed PMID: 22748516.

Beigelman A, Isaacson-Schmid M, Sajol G, Baty J, Rodriguez OM, Leege E, Lyons K, Schweiger TL, Zheng J, Schechtman KB, Castro M, Bacharier LB. Randomized trial to evaluate azithromycin's effects on serum and upper airway IL-8 levels and recurrent wheezing in infants with respiratory syncytial virus bronchiolitis. J Allergy Clin Immunol. 2015 May;135(5):1171-8.e1. doi: 10.1016/j.jaci.2014.10.001. Epub 2014 Nov 18. PubMed PMID: 25458910; PubMed Central PMCID: PMC4426225.

McCallum GB, Morris PS, Chatfield MD, Maclennan C, White AV, Sloots TP, Mackay IM, Chang AB. A single dose of azithromycin does not improve clinical outcomes of children hospitalised with bronchiolitis: a randomised, placebo-controlled trial. PLoS One. 2013 Sep 25;8(9):e74316. doi: 10.1371/journal.pone.0074316. eCollection 2013. PubMed PMID: 24086334; PubMed Central PMCID: PMC3783434.

Kabir AR, Mollah AH, Anwar KS, Rahman AK, Amin R, Rahman ME. Management of bronchiolitis without antibiotics: a multicentre randomized control trial in Bangladesh. Acta Paediatr. 2009 Oct;98(10):1593-9. doi: 10.1111/j.1651-2227.2009.01389.x. Epub 2009 Jul 1. PubMed PMID: 19572992.

Kneyber MC, van Woensel JB, Uijtendaal E, Uiterwaal CS, Kimpen JL; Dutch Antibiotics in RSV Trial (DART) Research Group. Azithromycin does not improve disease course in hospitalized infants with respiratory syncytial virus (RSV) lower respiratory tract disease: a randomized equivalence trial. Pediatr Pulmonol. 2008 Feb;43(2):142-9. PubMed PMID: 18085694.

Heliox

**Systematic reviews, n = 1**

Liet JM, Ducruet T, Gupta V, Cambonie G. Heliox inhalation therapy for bronchiolitis in infants. Cochrane Database Syst Rev. 2015 Sep 18;(9):CD006915. doi: 10.1002/14651858.CD006915.pub3. Review. PubMed PMID: 26384333.

**RCTs, n = 6**

Chowdhury MM, McKenzie SA, Pearson CC, Carr S, Pao C, Shah AR, Reus E, Eliahoo J, Gordon F, Bland H, Habibi P. Heliox therapy in bronchiolitis: phase III multicenter double-blind randomized controlled trial. Pediatrics. 2013 Apr;131(4):661-9. doi: 10.1542/peds.2012-1317. Epub 2013 Mar 18. PubMed PMID: 23509160.

Kim IK, Phrampus E, Sikes K, Pendleton J, Saville A, Corcoran T, Gracely E, Venkataraman S. Helium-oxygen therapy for infants with bronchiolitis: a randomized controlled trial. Arch Pediatr Adolesc Med. 2011 Dec;165(12):1115-22. doi: 10.1001/archpediatrics.2011.605. PubMed PMID: 22147778.

Iglesias Fernández C, Huidobro Fernández B, Míguez Navarro C, Guerrero Soler M, Vázquez López P, Marañón Pardillo R. [Heliox-driven bronchodilator nebulization in the treatment of infants with bronchiolitis]. An Pediatr (Barc). 2009 Jan;70(1):40-4. doi: 10.1016/j.anpedi.2008.08.001. Epub 2008 Nov 11. Spanish. PubMed PMID: 19174118.

Cambonie G, Milési C, Fournier-Favre S, Counil F, Jaber S, Picaud JC, Matecki S. Clinical effects of heliox administration for acute bronchiolitis in young infants. Chest. 2006 Mar;129(3):676-82. PubMed PMID: 16537867.

Hollman G, Shen G, Zeng L, Yngsdal-Krenz R, Perloff W, Zimmerman J, Strauss R. Helium-oxygen improves Clinical Asthma Scores in children with acute bronchiolitis. Crit Care Med. 1998 Oct;26(10):1731-6. PubMed PMID: 9781732.

Liet JM, Millotte B, Tucci M, Laflammme S, Hutchison J, Creery D, Ducruet T, Lacroix J; Canadian Critical Care Trials Group. Noninvasive therapy with helium-oxygen for severe bronchiolitis. J Pediatr. 2005 Dec;147(6):812-7. PubMed PMID: 16356437.

Oxygen Therapy

**Systematic reviews, n = 1**

Beggs S, Wong ZH, Kaul S, Ogden KJ, Walters JA. High-flow nasal cannula therapy for infants with bronchiolitis. Cochrane Database Syst Rev. 2014 Jan 20;(1):CD009609. doi: 10.1002/14651858.CD009609.pub2. Review. PubMed PMID: 24442856.

**RCTs, n = 5 (all included studies for this intervention)**

Kepreotes E, Whitehead B, Attia J, Oldmeadow C, Collison A, Searles A, Goddard B, Hilton J, Lee M, Mattes J. High-flow warm humidified oxygen versus standard low-flow nasal cannula oxygen for moderate bronchiolitis (HFWHO RCT): an open, phase 4, randomised controlled trial. Lancet. 2017 Mar 4;389(10072):930-939. doi: 10.1016/S0140-6736(17)30061-2. Epub 2017 Feb 2. PubMed PMID: 28161016.

Milési C, Essouri S, Pouyau R, Liet JM, Afanetti M, Portefaix A, Baleine J, Durand S, Combes C, Douillard A, Cambonie G; Groupe Francophone de Réanimation et d’Urgences Pédiatriques (GFRUP). High flow nasal cannula (HFNC) versus nasal continuous positive airway pressure (nCPAP) for the initial respiratory management of acute viral bronchiolitis in young infants: a multicenter randomized controlled trial (TRAMONTANE study). Intensive Care Med. 2017 Feb;43(2):209-216. doi: 10.1007/s00134-016-4617-8. Epub 2017 Jan 26. PubMed PMID: 28124736.

Bueno Campaña M, Olivares Ortiz J, Notario Muñoz C, Rupérez Lucas M, Fernández Rincón A, Patiño Hernández O, Calvo Rey C. High flow therapy versus hypertonic saline in bronchiolitis: randomised controlled trial. Arch Dis Child. 2014 Jun;99(6):511-5. doi: 10.1136/archdischild-2013-305443. Epub 2014 Feb 12. PubMed PMID: 24521787.

Hilliard TN, Archer N, Laura H, Heraghty J, Cottis H, Mills K, Ball S, Davis P. Pilot study of vapotherm oxygen delivery in moderately severe bronchiolitis. Arch Dis Child. 2012 Feb;97(2):182-3. doi: 10.1136/archdischild-2011-301151. Epub 2011 Nov 18. PubMed PMID: 22100741.

Milési C, Matecki S, Jaber S, Mura T, Jacquot A, Pidoux O, Chautemps N, Novais AR, Combes C, Picaud JC, Cambonie G. 6 cmH2O continuous positive airway pressure versus conventional oxygen therapy in severe viral bronchiolitis: a randomized trial. Pediatr Pulmonol. 2013 Jan;48(1):45-51. doi: 10.1002/ppul.22533. Epub 2012 Mar 19. PubMed PMID: 22431446.

Hypertonic Saline

**Systematic reviews, n = 1**

Maguire C, Cantrill H, Hind D, Bradburn M, Everard ML. Hypertonic saline (HS) for acute bronchiolitis: Systematic review and meta-analysis. BMC Pulm Med. 2015 Nov 23;15:148. doi: 10.1186/s12890-015-0140-x. Review. PubMed PMID: 26597174; PubMed Central PMCID: PMC4657365.

**RCTs, n = 6**

Köse S, Şehriyaroğlu A, Esen F, Özdemir A, Kardaş Z, Altuğ U, Karakuş E, Özcan A, Kısaarslan AF, Elmalı F, Torun YA, Köse M. Comparing the Efficacy of 7%, 3% and 0.9% Saline in Moderate to Severe Bronchiolitis in Infants. Balkan Med J. 2016 Mar;33(2):193-7. doi: 10.5152/balkanmedj.2016.16840. Epub 2016 Mar 1. PubMed PMID: 27403389; PubMed Central PMCID: PMC4924964.

Flores P, Mendes AL, Neto AS. A randomized trial of nebulized 3% hypertonic saline with salbutamol in the treatment of acute bronchiolitis in hospitalized infants. Pediatr Pulmonol. 2016 Apr;51(4):418-25. doi: 10.1002/ppul.23306. Epub 2015 Sep 3. PubMed PMID: 26334188.

Angoulvant F, Bellêttre X, Milcent K, Teglas JP, Claudet I, Le Guen CG, de Pontual L, Minodier P, Dubos F, Brouard J, Soussan-Banini V, Degas-Bussiere V, Gatin A, Schweitzer C, Epaud R, Ryckewaert A, Cros P, Marot Y, Flahaut P, Saunier P, Babe P, Patteau G, Delebarre M, Titomanlio L, Vrignaud B, Trieu TV, Tahir A, Regnard D, Micheau P, Charara O, Henry S, Ploin D, Panjo H, Vabret A, Bouyer J, Gajdos V; Efficacy of 3% Hypertonic Saline in Acute Viral Bronchiolitis (GUERANDE) Study Group. Effect of Nebulized Hypertonic Saline Treatment in Emergency Departments on the Hospitalization Rate for Acute Bronchiolitis: A Randomized Clinical Trial. JAMA Pediatr. 2017 Aug 7;171(8):e171333. doi: 10.1001/jamapediatrics.2017.1333. Epub 2017 Aug 7. PubMed PMID: 28586918.

Wu S, Baker C, Lang ME, Schrager SM, Liley FF, Papa C, Mira V, Balkian A, Mason WH. Nebulized hypertonic saline for bronchiolitis: a randomized clinical trial. JAMA Pediatr. 2014 Jul;168(7):657-63. doi: 10.1001/jamapediatrics.2014.301. Erratum in: JAMA Pediatr. 2014 Oct;168(10):971. PubMed PMID: 24862623.

Teunissen J, Hochs AH, Vaessen-Verberne A, Boehmer AL, Smeets CC, Brackel H, van Gent R, Wesseling J, Logtens-Stevens D, de Moor R, Rosias PP, Potgieter S, Faber MR, Hendriks HJ, Janssen-Heijnen ML, Loza BF. The effect of 3% and 6% hypertonic saline in viral bronchiolitis: a randomised controlled trial. Eur Respir J. 2014 Oct;44(4):913-21. doi: 10.1183/09031936.00159613. Epub 2014 Jun 25. PubMed PMID: 24969648.

Everard ML, Hind D, Ugonna K, Freeman J, Bradburn M, Cooper CL, Cross E, Maguire C, Cantrill H, Alexander J, McNamara PS; SABRE Study Team. SABRE: a multicentre randomised control trial of nebulised hypertonic saline in infants hospitalised with acute bronchiolitis. Thorax. 2014 Dec;69(12):1105-12. doi: 10.1136/thoraxjnl-2014-205953. PubMed PMID: 25389139; PubMed Central PMCID: PMC4251206.

**Search strategy (Pubmed Similar Articles)**

**Database:** PubMed via NCBI Entrez

**Date of search:** Monthly

| Search #1: Test set of seed articles  29158779[uid] or 26037525[uid] or 12840097[uid] or 14757604[uid] or 21471175[uid] or 24937099[uid] or 25263242[uid] or 26478870[uid] or 24687251[uid] or 19439742[uid] or 18370982[uid] or 23758233[uid] or 24381968[uid] or 10742365[uid] or 23733383[uid] or 27097575[uid] or 25129681[uid] or 19445861[uid] or 17652648[uid] or 24043283[uid] or 17394255[uid] or 25300167[uid] or 17050564[uid] or 22748516[uid] or 25458910[uid] or 24086334[uid] or 19572992[uid] or 18085694[uid] or 26384333[uid] or 23509160[uid] or 22147778[uid] or 19174118[uid] or 16537867[uid] or 9781732[uid] or 16356437[uid] or 24442856[uid] or 28161016[uid] or 28124736[uid] or 24521787[uid] or 22100741[uid] or 22431446[uid] or 26597174[uid] or 27403389[uid] or 26334188[uid] or 28586918[uid] or 24862623[uid] or 24969648[uid] or 25389139[uid] (48)  Search #2: Similar articles  Find related data > Database: PubMed  Option: Similar articles [Similar PubMed articles, obtained by matching text and MeSH terms]  Search #3: Date range limit (using create date field for each month)  #2 AND ("2018/09/01"[CRDT]: "2018/09/30"[CRDT]) |
| --- |

**Appendix 5.** Screening data by month

| **Date of search** | **Search approach** | **Records retrieved and screened by title and abstract** | **Records screened by full text** | **Eligible trials**^a^ | **Included trials**^b^ |
| --- | --- | --- | --- | --- | --- |
| Sep-18 | Automated full search | 62 | 1 | 1 | 1 |
|  | Citing References (Scopus) | 15 | 0 | 0 | 0 |
|  | Similar Articles (PubMed) | 33 | 1 | 0 | 0 |
| Oct-18 | Automated full search | 57 | 0 | 0 | 0 |
|  | Citing References (Scopus) | 27 | 0 | 0 | 0 |
|  | Similar Articles (PubMed) | 36 | 1 | 0 | 0 |
| Nov-18 | Automated full search | 39 | 1 | 0 | 0 |
|  | Citing References (Scopus) | 36 | 1 | 0 | 0 |
|  | Similar Articles (PubMed) | 37 | 1 | 0 | 0 |
| Dec-18 | Automated full search | 55 | 1 | 0 | 0 |
|  | Citing References (Scopus) | 49 | 2 | 0 | 0 |
|  | Similar Articles (PubMed) | 24 | 0 | 0 | 0 |
| Jan-19 | Automated full search | 62 | 4 | 1 | 1 |
|  | Citing References (Scopus) | 29 | 1 | 0 | 0 |
|  | Similar Articles (PubMed) | 43 | 1 | 0 | 0 |
| Feb-19 | Automated full search | 54 | 2 | 0 | 0 |
|  | Citing References (Scopus) | 11 | 1 | 0 | 0 |
|  | Similar Articles (PubMed) | 29 | 1 | 0 | 0 |
| Mar-19 | Automated full search | 72 | 4 | 3 | 2 |
|  | Citing References (Scopus) | 9 | 0 | 1 | 1 |
|  | Similar Articles (PubMed) | 44 | 2 | 0 | 0 |
| Apr-19 | Automated full search | 87 | 1 | 0 | 0 |
|  | Citing References (Scopus) | 15 | 0 | 0 | 0 |
|  | Similar Articles (PubMed) | 39 | 0 | 0 | 0 |
| May-19 | Automated full search | 98 | 4 | 2 | 0 |
|  | Citing References (Scopus) | 24 | 1 | 0 | 0 |
|  | Similar Articles (PubMed) | 44 | 0 | 1 | 0 |
| Jun-19 | Automated full search | 87 | 2 | 0 | 0 |
|  | Citing References (Scopus) | 2 | 1 | 0 | 0 |
|  | Similar Articles (PubMed) | 38 | 1 | 0 | 0 |
| Jul-19 | Automated full search | 68 | 1 | 0 | 0 |
|  | Citing References (Scopus) | 13 | 0 | 0 | 0 |
|  | Similar Articles (PubMed) | 43 | 1 | 0 | 0 |
| Aug-19 | Automated full search | 75 | 0 | 0 | 0 |
|  | Citing References (Scopus) | 14 | 0 | 0 | 0 |
|  | Similar Articles (PubMed) | 42 | 2 | 0 | 0 |
| **TOTAL (1-year)** | **Automated full search** | **816** | **21** | **7** | **4** |
|  | **Citing References (Scopus)** | **244** | **7** | **1** | **1** |
|  | **Similar Articles (PubMed)** | **452** | **11** | **1** | **0** |

^a^ Eligible trials were those that met the eligibility criteria for the SR.

^b^ Included studies were those that met the eligibility criteria and had not been previously located by another (or the same) search approach. One study (Chen 2019) was located in the same month by the automated full search and the Scopus Cited References search.

**Appendix 6.** Characteristics of the four new included trials

| **Study**  **Design**  **Funding source** | **Country**  **Setting** | **Population** | **Intervention & Comparator** | **Outcomes** |
| --- | --- | --- | --- | --- |
| Chen 2019 [23]  RCT (2-arm parallel)  Industry | United States  Inpatient, single centre | 32 children aged 0-12 months with moderate bronchiolitis | I: Heated and humidified oxygen at high flow via nasal cannula  C: Standard dry oxygen at low flow via nasal cannula | Length of stay |
| Ergul 2018 [24]  RCT (2-arm parallel)  Not reported | Turkey  Inpatient, single centre | 60 children aged 1-24 months with moderate to severe bronchiolitis | I: High flow oxygen via nasal cannula  C: High flow oxygen via diffuser mask | Length of stay |
| Morikawa 2018 [25]  RCT (2-arm parallel)  No external funding | Japan  Inpatient, 5 centres | 128 children aged <12 months with mild to moderate bronchiolitis due to RSV | I: 0.1 ml of 0.5% salbutamol in 2 ml of 3% nebulized hypertonic saline  C: 0.1 ml of 0.5% salbutamol in 2 ml of 0.9% nebulized saline  + oxygen in both groups if SpO_2_ <95% | Length of stay |
| Seliem 2019 [26]  RCT (2-arm parallel)  Not reported | Egypt  Inpatient, single centre | 48 children aged 1 month-2 years with moderate to severe bronchiolitis due to RSV | I: Heliox (79:21) at low flow via nasal cannula  C: Air oxygen at low flow via nasal cannula | No primary outcomes presented |

C=comparator; I=intervention; RSV=respiratory syncytial virus; SpO_2_=oxygen saturation level

**Appendix 7.** Forest plots for the analysis of length of stay

**Oxygen therapy vs. control**

Baseline (August 2018)


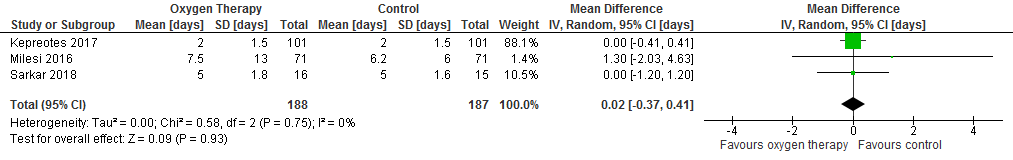


CI=confidence interval; IV=inverse variance; SD=standard deviation

March 2019 (added Chen 2019 and Ergul 2018)


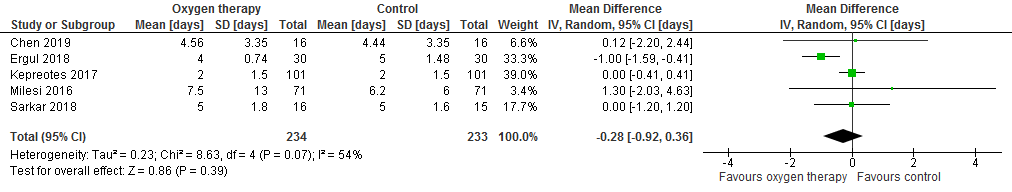


CI=confidence interval; IV=inverse variance; SD=standard deviation

**Hypertonic saline vs. control**

Baseline (August 2018)


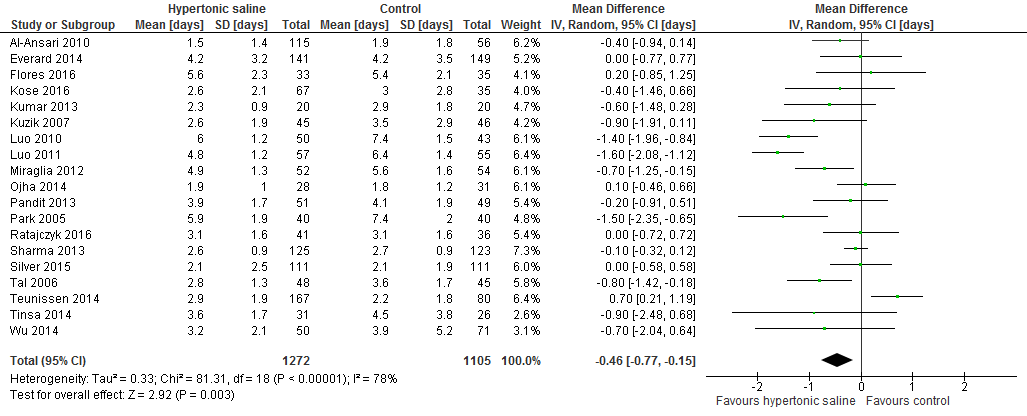
CI=confidence interval; IV=inverse variance; SD=standard deviation

September 2018 (added Morikawa 2018)


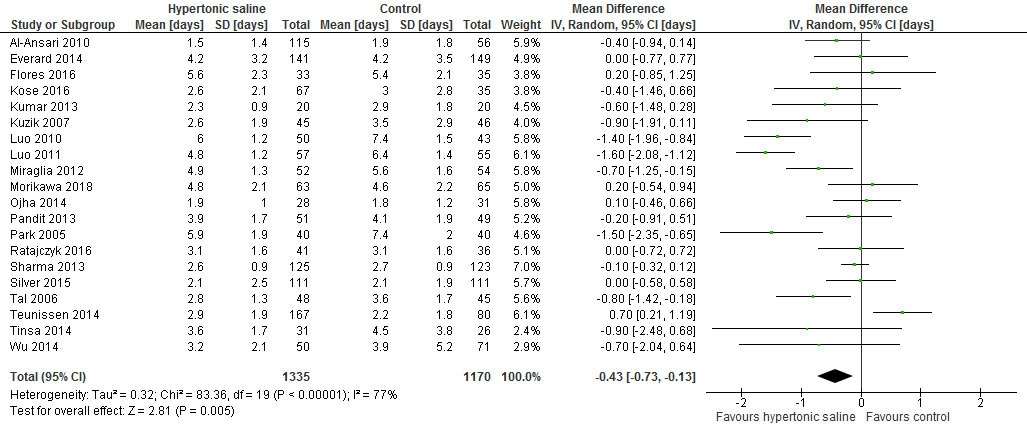


CI=confidence interval; IV=inverse variance; SD=standard deviation
